# The Robot-Assisted Extravesical Anti-reflux Surgery: How We Overcame the Learning Curve

**DOI:** 10.3389/fped.2019.00093

**Published:** 2019-03-29

**Authors:** Ravindra Sahadev, Katelyn Spencer, Arun K. Srinivasan, Christopher J. Long, Aseem Ravindra Shukla

**Affiliations:** Division of Pediatric Urology, Children's Hospital of Philadelphia, Philadelphia, PA, United States

**Keywords:** robotic, ureteral reimplantation, RALUR, learning curve, extravesical approach

## Abstract

Management of vesicoureteral reflux (VUR) has evolved over the past several decades, with a trend toward a decrease in surgical management. In spite of this, ureteral reimplantation remains a commonly performed procedure by pediatric urologists in selected cases. Although the basic tenets of the ureteral reimplant procedure remain the same, the extra- vs. intravesical approach, and the traditional open vs. minimally invasive approach remain the primary options to correct reflux. Considering the advantages conferred by the robotic surgery platform, many leading centers have preferentially adopted robot-assisted laparoscopic extravesical anti-reflux surgery, or in common surgical parlance, the robot-assisted laparoscopic ureteral reimplantation (RALUR), over pure laparoscopic or open approaches. Predicated on our experience of performing over 170 cases of RALUR, we have made technical modifications which we posit reduce the morbidity of the procedure while offering acceptable outcomes. This review highlights the evolution and establishment of RALUR as a standardization of care in the surgical management of VUR at our institution. In particular, we emphasize the technical nuances and specific challenges encountered through the learning curve in hopes of facilitating this process for others.

## Introduction

Over the past two decades, both the evaluation and therapeutic interventions for vesicoureteral reflux (VUR) have undergone extensive evolutions, with a clear trend toward non-operative management and a focus on voiding dysfunction as the major risk factor for urinary tract infection (UTI) (1–3).

In spite of significant investigations aimed at identifying risk factors for the development of recurrent urinary tract infections and predicting the potential for VUR resolution and/or the development of renal scarring, our ability to do so remains limited. Still, when conservative management fails, and febrile UTIs or significant renal scarring occurs, children do require surgical management.

The surgical principles for the correction of VUR remain consistent, several years after its initial description (4). In order to prevent VUR the length of the anti-refluxing tunnel is extended, traditionally in a 5:1 ratio of tunnel length to width of the ureter. While the open ureteral reimplant has been the traditional gold standard repair for VUR, the minimally invasive approaches, such as sub-ureteral injections or laparoscopic and robotic assisted laparoscopic ureteral reimplant (RALUR), have been established as viable alternatives (3).

Although initial enthusiasm for RALUR has waned for some due to concerns about a steep learning curve which can potentially increase the risk of patient morbidity or persistent VUR (5, 6), the well-established benefits of the robotic approach encourage its use (7, 8). Benefits of the robotic vs. the open approach include an improved field of vision via magnification intraperitoneal visualization of the ureters and bladder, improved cosmesis for the patient, and a more rapid recovery in the immediate postoperative period due to an extravesical approach. In our experience several technical modifications mitigate the aforementioned risk of RALUR while maximizing patient outcomes.

In this review, we seek to explore the evolution of extravesical RALUR–as it is the most widely adapted robotic approach–and describe technical modifications that render this procedure reproducible across surgeons and lessen the learning curve trajectory.

## Evolution of RALUR

Since its first description by Peters (9), the technical aspects of the RALUR have undergone several modifications. Along with the initial descriptions of robotic assisted pediatric urologic procedures, Peters highlighted the advantages of the robotic system, the need for evolution and evaluation (9). The surgical principles illustrated for both the robotic-assisted intravesical (RAIVUR) and extravesical techniques were adapted to the new platform while adhering to the principles of contemporary open surgical procedures. The intravesical techniques were favored in bilateral cases due the concerns of urinary retention with bilateral extravesical reimplantation (9).

Since then, while the RAIVUR confers advantages of decreased hematuria and bladder spasms compared to the open approach, it failed to gain widespread adaptation, largely due to technical challenges such as insufflation leaks through the larger trocar hiatus and limited working space of a small bladder (10, 11). Only three groups have reported their experience with RAIVUR, with modest success rates (83, 92, and 100% reflux resolution in 6, 19, and 3 patients, respectively) and highly variable complication rates (17, 52, and 0%) (10–12).

Meanwhile, the Lich-Gregoir technique has become popular for the robotic approach due to its ready adaptability to the technology. This trans-abdominal approach provides excellent visualization of the retrovesical space, particularly when compared to the open approach. The magnification of the robotic camera facilitates meticulous detrusor dissection, which combined with judicious use of energy devices limits the potential for collateral damage to the nerve bundles of the bladder (13).

**Figure 1:**
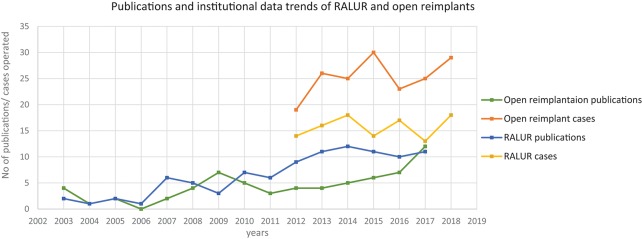
Trends in publications and number of RALURs performed at our institute. Source: PubMed search report of terms “Open ureteral reimplantation” and “Robot-assisted ureteral reimplantation” as on 23 July 2018.

As with other minimally invasive approaches, RALUR confers the significant benefits of minimally invasive surgery including a shorter convalescence, reduced hospital stay, and improved cosmesis. The realization of the potential improved experience for patients has led to more widespread acceptance for utilization of RALUR. This had led to more centers having a higher number of RALUR over open ureteral reimplants, including ours, and this is reflected in publication trends as well (Figure 1).

A negative postoperative voiding cystourethrogram (VCUG) remains the gold standard to declare surgical success after a ureteral reimplantation. But, traditionally high success rates of the open approach obviated a post-operative VCUG in practice. Similarly, with accumulating experience with the RALUR, and pilot studies have demonstrated that our technique delivers reliable VCUG proven success (14). Our institution now defines surgical success as a lack of postoperative febrile UTI and a negative VCUG, if obtained in the postoperative period (15). Potential short term complications reported after RALUR include minor self-limiting adverse events such as bladder spasms, hematuria, and GI disturbances. Other reported complications include urinary extravasation, UTIs, incisional hernia, ureteral obstruction resulting in anuria, and the rare complication of ureteral strictures.

Early reports for RALUR are typical for most new techniques: they are comprised of single institution experiences with small patient numbers. To compound this, there is a lack of uniformity in data reporting, including the age at the time of repair, the indication for ureteral reimplantation (VUR vs. obstruction), and the degree of reflux at the time of surgery. Bladder and bowel dysfunction has proven to be a significant risk factor for surgical failure yet remains under reported in the literature (16). Overview of the literature review (Table 1) suggests, in the first decade of utilization of RALUR, there were inconsistencies in reported success (66.7–100%) and complication (0–100%) rates (7–9, 11–15, 17–28). It is notable that higher Clavien grade complications occurred in reports following smaller patient cohorts. However, recent growing evidence in literature has been relatively consistent in proving the safety and efficacy of RALUR in prospective multi-institutional collaborative efforts (7, 8).

**Table 1:** A literature review of RALUR-EV.

**References**	**Study year^†^**	**Baseline**			**Outcomes**			
		**No of patients**	**No of Ureters**	**OR time in min**	**Success rate^#^**	Complications %^*^		
						**Clavien grade 1**	**Clavien grade 2**	**Clavien grade 3**
Peters (9)	2004	24	27	165	88		11	5
Chan et al. (12)	2005–2008	3	4	210	66.7	33	0	0
Boysen et al. (7)	2005–2014	260	363	177	87.9	5	1.9	2.7
Casale et al. (13)	2006–2007	41	82	139.8	97.6	0	4.87	0
Smith et al. (17)	2006–2009	25	33	185	95.6	16	0	4
Akhavan et al. (18)	2006–2013	50	78	–	92.3	4	14	4
Chalmers et al. (19)	2007–2010	16	22	152	90.9	0	0	0
Marchini et al. (11) (EV)	2007–2010	20	27	233.5	100	10	10	0
Schomburg et al. (14)	2008–2010	20	25	196	100	0	0	10
Gundeti et al. (20)	2008–2015	58	83	–	82	1.7	0	0
Silay et al. (21)	2008–2015	72	91	–	97.9	2.7	0	0
Katsturi et al. (22)	2009–2011	150	300	–	99.3	0	0.7	0
Dangle et al. (23)	2010–2013	29	40	–	80	–	–	–
Grimsby et al. (24)	2010–2013	61	93	–	72	1.6	1.6	8.2
Srinivasan et al. (15)	2012–2016	92	–	164	91.3	4.3	17	2.1
Hayashi et al. (25)	2013	9	15	268.78	93.3	100	0	0
Arlen et al. (26)	2013–2014	17	20	169.3	94.1	0	11.76	0
Herz et al. (27)	2013–2015	54	72	–	85.2	5.5	9.8	11
Boysen et al. (8)	2015–2017	143	199	194	93.8	4.9	0.7	5.6

† *Approximate period*.

# *Clinical or radiological success rates*.

* *Some reports did not report complications as per Clavien-Dindo grading directly; The reports were graded based on the description. Some authors included UTIs in the complications. There were no Grade-4 complications related to surgery reported in any of the studies. In many series, same patients have had complications of different grades, hence they cannot be summated*.

## The Learning Curve

The learning curve refers to variations in the productivity of a new surgical procedure or surgeon over a specific time period that leads to achieving a consistent level of expertise to meet contemporary standards. Defining a learning curve is a challenging concept. Although some authors claim that proxies such as operative time, complication rates, and functional outcomes are inadequate measures to assess a true learning curve, most reports have defaulted to these as practical measures of surgical success (28, 29).

As outlined above, the heterogeneity of the published literature on RALUR on key variables such as patient demographics, grades of reflux, comorbidities, in addition to variable study catchment periods makes assessment of a true learning curve difficult.

In order to trace the influence of a learning curve to surgical parameters and outcomes for RALUR at our institution, we have compared the failure rates (need for secondary anti-reflux surgery), radiographic reflux resolution rate, operative time, and/or complication rates over defined time frames (yearly trends) for a series of consecutive cases (Table 2). We feel as though a single institution center with a relatively high surgical volume as well as 5 different surgeons provides a unique opportunity to carry out this analysis.

**Table 2:** Summary of institutional RALUR data: patient characteristics and operative outcomes.

**Year**	**No of pts**.	**Age in yrs**.	**No of renal units**	**VUR- I, II, III, IV, V, OMs**	**OR time in min**	**Need for subsequent anti-reflux surgery**	**Complications-Grade- 1, 2, 3,4**	**Mean follow up (months)**
2012	15	4.53	18	0,3,3,7,1,1	132	1	3,1,1,0	23.36
2013	34	5.39	41	0,5,12,7,2,8	174	0	2,1,3,0	32.76
2014	25	5.18	30	3,2,9,5,3,3	165	1	0,1,1,0	33.43
2015	29	5.31	43	0,3,10,11,2,3	194	3	1,1,3,0	25.99
2016	24	7.62	38	0,4,8,7,2,3	206	0	1,0,1,0	20.35
2017	22	6.25	31	0,3,9,3,2,5	203	0	0,0,0,0	11.75
2018^#^	21	5.52	29	0,4,6,4,4,3	197	0	1,0,0,0	3.57

*OM-Obstructive megaureter. # till October 2018*.

**Figure 2:**
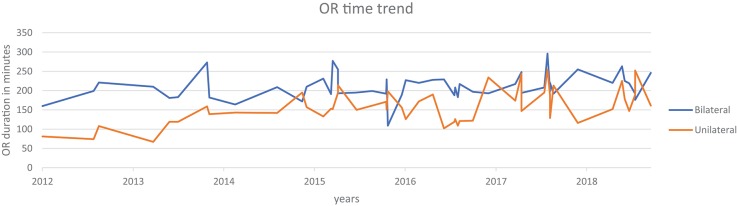
Institutional- OR time trends over 2012 to 2018.

A review of data from a prospectively maintained database from 2012 till October 2018, includes six surgeons performing RALUR on 170 patients, of which 60 were bilateral. One hundred twenty-seven were female patients. In our series, the average age at surgery was 5.9 years with a general trend toward higher age at surgery each year, contrary to the literature on national data (3). A Majority of RALUR were done for dilating VUR (Grade 3 or higher) with breakthrough UTIs or renal scarring (100 cases), 36 cases had a duplex anomaly, 23 obstructive megaureter and 10 bladder diverticula. Additionally, 18 cases had a prior history of failed sub-ureteric injection. Among VUR cases, 40% had high grade VUR (IV and V). The operative time varied depending on the number of ureters, need for cystoscopy, retrograde pyelography, placement of a suprapubic tube, and other concomitant procedures depending on the associated pathology (nephrectomy, heminephrectomy, etc.). For unilateral procedures without any concomitant procedure the mean operative time was 161 min (49 cases), and it was 208 min for bilateral cases (48 cases). Operative time includes time of first incision or procedure start, to procedure end time as recorded by the nursing staff. The operative time did not vary significantly between the first and last quarters of consecutive case series (Figure 2). Blood loss was minimal in most of the cases from the beginning.

Over the follow up period of 1 to 75 months (mean 23, median 20 months), six cases had transient urinary retention and four cases needed surgery for port site hernia (we now meticulously close fascia even for 5 mm port sites under direct vision). Four cases of ureteral obstruction were noted based on increased dilation of calyces on renal ultrasound with symptoms of flank pain with nausea; of which three cases resolved with cystoscopy and ureteral stenting for 6 weeks and, in another case, required open ureteral reimplantation. We previously reported our surgical outcomes on an initial cohort and found that postoperative febrile UTIs occurred in 15.8% of unilateral cases compared with 20% of bilateral cases (*p* = 0.61) (15). Surgical failure, denoted by postoperative febrile UTI and a positive VCUG was noted in five (8.7%) of the unilateral cases vs. three (8.6%) of the bilateral RALUR cases (*p* = 0.98). The updated demographic and clinical information is summarized in Table 2.

Although the intraoperative surgical time has remained consistent over the years, we note that the number and complexity of complications are decreasing over subsequent years, concomitant to a reduced need for secondary interventions. Indeed, these results encouraged increasing use of RALUR at our institution with the increase in expertise and confidence level. Backed by comparable outcomes there has been an increase in the proportion of RALUR cases as compared to open ureteral reimplants (Figure 1). We expect, also, that being an academic teaching institute with many surgeons and trainees, inherent variations in the proficiency levels with atypical learning curve patterns would affect continued improvement in specific parameters.

## Technical Modifications to Achieve a Successful RALUR

Based on our experiences with RALUR, in addition to standard steps (Table 3), we have adopted several key technical modifications that we believe have improved our institutional outcomes (Table 4). Proper case selection is vital and must consider patient age, toilet training status, and the presence or absence of dysfunctional elimination. If there is a concern for secondary reflux due to a neurogenic bladder, this must be worked up prior to intervention. If significant bowel and/or bladder dysfunction persists in spite of adequate therapy, a suprapubic tube should be considered in order to ensure proper, low-pressure post-operative voiding prior to removing within a week.

**Table 3:** Standard steps of RALUR.

•Preoperative cystoscopy and retrograde pyelogram, if there is a suspicion of ectopic ureter, duplex systems, solitary system •Patient position: Supine (younger patients) or modified lithotomy; Attention to pressure points and careful strapping to prevent shifting when patient put into lithotomy position. •Port placement: 8.5 mm camera port at the umbilicus, 5 mm working instruments in midclavicular line on either side, or utilizing lower incisions with cephalad tunneling of the trocar. •Cephalad ureteral mobilization to release tension •Tunnel measurement and marking to achieve a 5:1 ratio •Mild hydrodistension, hitch placement •Detrusor tunnel creation •Detrusorrhaphy-suturing •Closure

**Table 4:** Challenges and cautions/modifications.

**Outcomes of concerns**	**Possible technical reasons**	**Modifications adopted**
Urinary retention	Damage to nerve plexus	Precise dissection at VUJ between ureter and bladder, avoiding medial and caudal detrusor dissection (Figure 4C)
		Judicious use of electrocautery
Incomplete reflux resolution	Inadequate tunnel length	Standardized measurement of the tunnel length in the collapsed bladder (Figure 3C)
	Cephalad slippage of the ureter out of the tunnel	Including the ureter adventitia in the first few and last tunnel closure suture (“advancement stitches”). Proximal ureteral mobilization to release tension on tunnel closure
	Incomplete detrusor separation for the tunnel creation	Use of hitch stitch for adequate traction and bladder distension to facilitate dissection (Figure 3D)
Ureteral obstruction	Ureteral injury	Avoiding pre-stenting unless absolutely necessary (e.g., Duplex system), avoiding excessive traction and direct cautery usage on the ureter
	Excessive ureteral mobilization	Ureteral mobilization to the required length with frequent assessments
	Tight tunnel	“Bottom-up” approach starting at the UVJ with careful and stepwise closure of tunnel, raising adequate detrusor flaps to have a spacious tunnel
	Acute angulation of the ureter	Studying the course of the ureter and its angulation prior to hitch stitch and marking the corresponding tunnel line (Figure 3C)
Urinary leak	Cystotomy	Careful identification and repair prior to closure of detrussorotomy
	Leak from ureteral suture-line	Maximize urinary drainage with bladder catheter and/or suprapubic tube, and place ureteral stent if necessary.
	Refluxing stumps in cases of ectopic ureteral insertion	Adequate exposure, resection of residual, and closure of the stump.
Multiple post site scars	Multiple port site scars	HidES (Hidden incision for endoscopic surgery) groin ports, hide umbilical camera port within umbilical crease; only two working 5 mm ports; no assist port.
Injury to vas and vessels	Poor field of vision	Preservation of uterine vessels; Under-vision dissection distal to the vas deferens. Starting the distal ureteral dissection with good hemostasis to maintain optimum visibility (Figure 3A).

**Figure 3:**
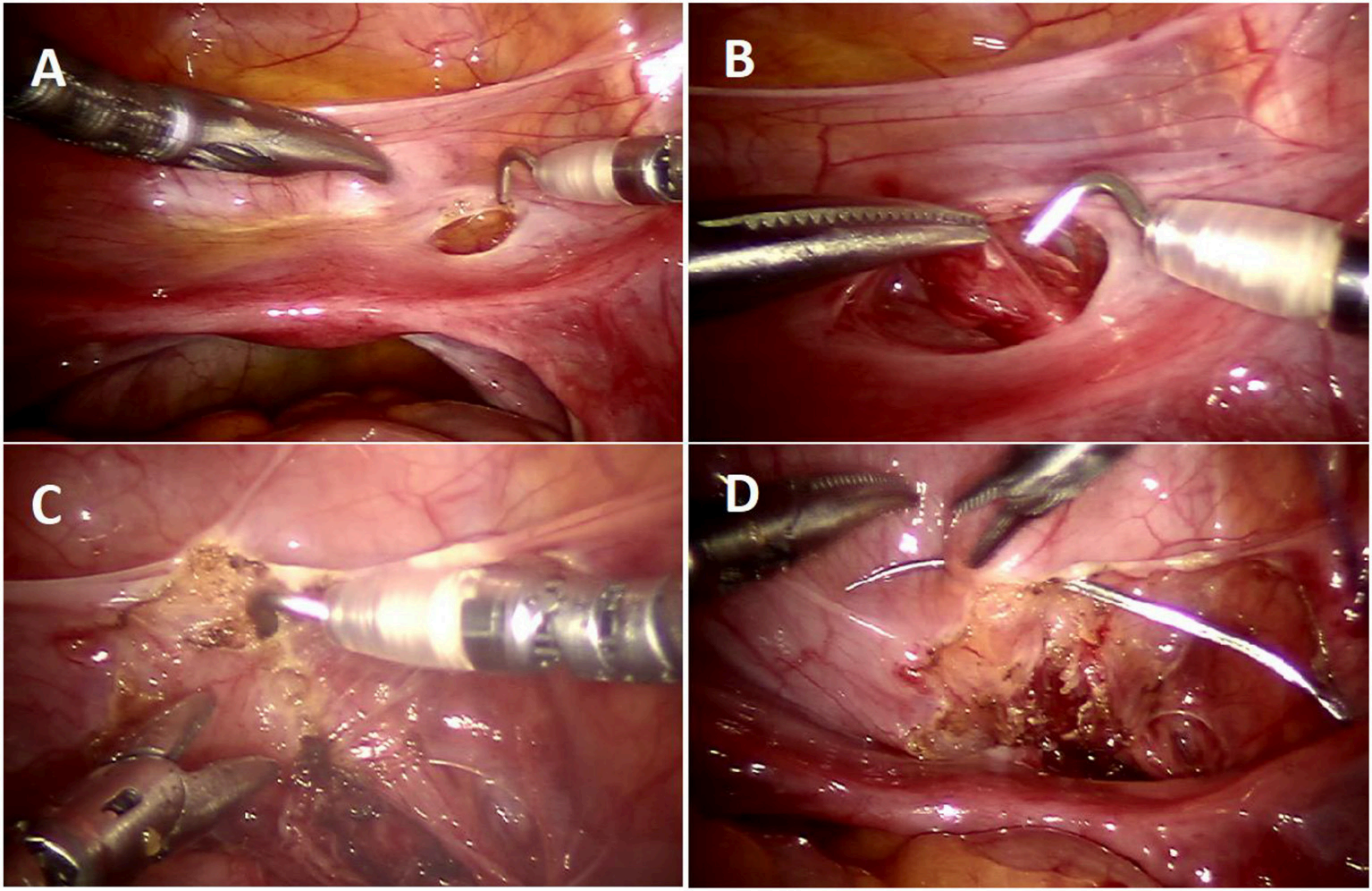
Operative steps of RALUR. **(A)** Small window created in the broad ligament to access the ureter directly. **(B)** Ureteral mobilization: gentle handling by grasping only the ureteral adventitia. **(C)** Marking the detrusor tunnel in a collapsed bladder in line with the ureter. **(D)** Hitch stitch at the distal end of tunnel marking.

**Figure 4:**
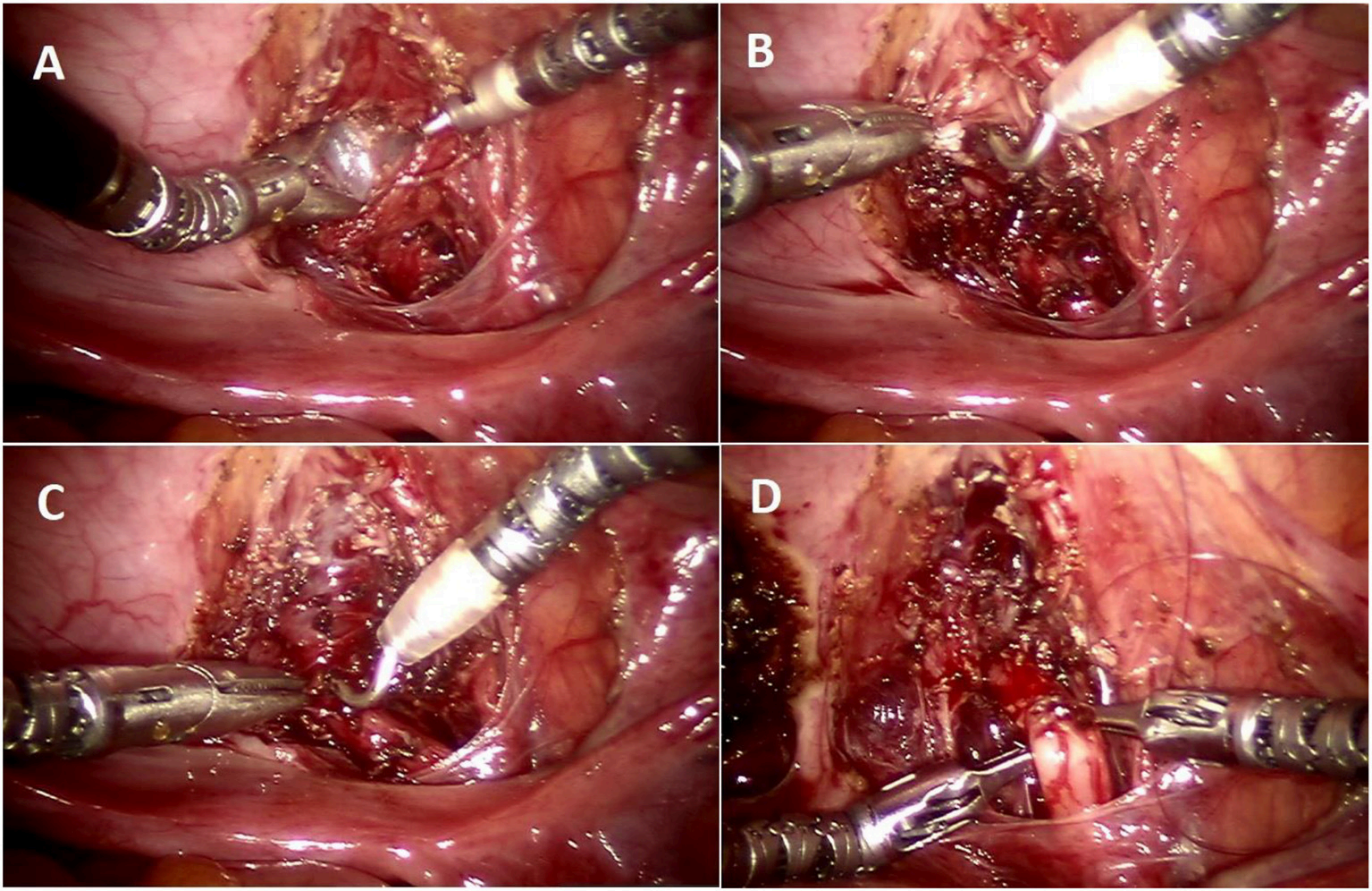
Operative steps of RALUR. **(A)** Making a detrusor window till bluish bladder mucosa is delineated. **(B)** Detrusor muscle separated with direct pinpoint electrocautery tip combined with blunt spreading of the muscle fibers. **(C)** Detrusor tunnel opened proximally to ureterovesical junction. **(D)** Passing suture underneath the ureter to advance the ureter into the detrusor trough (left to right).

In order to avoid collateral damage to the detrusor muscle and the nerve plexus, meticulous dissection and judicious use of electrocautery is vital (Figure 3B). We recommend that estimating tunnel length is inaccurate–and overestimated–when the bladder is even slightly distended, hence we now delineate the tunnel length–ensuring a measurement of tunnel length five times the diameter of the distal ureter–while the bladder is completely drained with a foley catheter (Figure 3C). We regularly utilize a hitch stitch not only to aid detrusor dissection but also to mark the direction of proposed tunnel (Figure 3D). We use only the tip of the hook to cauterize the identified bleeding spots and spread the muscles bluntly, rather than cutting those layers (Figure 4B).

Once the tunnel length of the detrusor is delineated, we begin dissecting the tunnel midway between the hitch stitch and ureterovesical junction until the bladder mucosal layer is defined (Figure 4A). Utilizing this window, we find that further progression proximally and distally proceeds more easily without the risk of inadvertent cystotomies (Figure 4C). We avoid medial and inferior dissection close to the VUJ, which poses damage to the nerve plexus and may increase the risk of detrusor injury and urinary retention. The ureters must be adequately mobilized in a cephalad direction in order to remove any proximal tension that may tease the ureter out of the tunnel.

Once the detrusorrhaphy begins, we prefer interrupted suturing with long term absorbable sutures (5–0 polydiaxone) in a “bottom up” approach, starting from uretero-vesical junction and then moving distally toward the hitch stitch (dome), as it allows for a tailored formation of the tunnel depending on the available ureter length and amount of tension. This technique can be confusing for the beginner and requires careful passing of suture underneath the ureter and back toward the initial bite side (Figure 4D) to allow placement of the ureter deep in the tunnel with close approximation of the detrusor edges. It is important to include ureteral adventitia in the initial and ending detrusor closure stitch. In cases of dismembered reimplants, solitary kidney, tapered or complex reimplants we prefer to leave a double J stent, but we do not stent typical RALUR cases. We also do not routinely place a drain. We leave a urinary catheter overnight and discharge the patient once they are able to void, leaving residuals that are <25% of the expected bladder capacity.

Table 4 summarizes the common pitfalls and technical modification adopted to address those concerns.

## Challenges

In their recent review, Baek et al. analyzes the reasons for slower adoption of RALUR in comparison to the widespread and quickly adopted robotic prostatectomy among adult counterparts. The steeper learning curve and concerns about the efficacy compared to open reimplants were oft-cited reasons, and the authors suggested that the procedure be deferred to a later point in the robotic experience (30). However, from our learning curve experience, we deduce that careful adherence to outlined steps allows the RALUR to be safely and reproducibly performed. Standardization of salient steps with adequate training and judicious use of electrocautery will ensure the avoidance of common pitfalls.

Although there are debates regarding the efficacy of the RALUR in comparison to open ureteral reimplantation, we must be cautious while comparing the historical reports to the contemporary outcomes as the population characteristics (age group, voiding dysfunction, etc.) have been changing. Eventually, the trends suggest RALUR inexorably will be adopted more widely, and reports and ongoing multi-institutional consortiums will continue to provide further evidence regarding its safety and affirming its efficacy.

## Conclusions

RALUR utilization has increased since its inception, but concerns over the procedure's technical difficulty, safety, risk of urinary retention and outcomes has limited its widespread use. Herein, we demonstrate that the learning curve of RALUR can be shortened with specific modifications predicated on experience, and that with technical adaptations, clinically significant improvements in surgical outcomes may be expected. In appropriately selected patients and with adequate preparation, we posit that the RALUR is a safe and effective technique that confers the well-described advantages of minimally invasive surgery to the treatment of VUR.

## Data Availability

All datasets generated for this study are included in the manuscript and/or the supplementary files.

## Author Contributions

ARS conceived the idea, laid the platform for the review with substantial additions to the manuscript, along with proofreading. RS prepared the initial manuscript and collected the raw data. KS prepared the literature review and part of data abstraction. CL and AKS reviewed the manuscript and made significant corrections.

### Conflict of Interest Statement

The authors declare that the research was conducted in the absence of any commercial or financial relationships that could be construed as a potential conflict of interest.
